# Use of Targeted High-Throughput Sequencing for Genetic Classification of Patients with Bleeding Diathesis and Suspected Platelet Disorder

**DOI:** 10.1055/s-0038-1676813

**Published:** 2018-12-30

**Authors:** Oliver Andres, Eva-Maria König, Karina Althaus, Tamam Bakchoul, Peter Bugert, Stefan Eber, Ralf Knöfler, Erdmute Kunstmann, Georgi Manukjan, Oliver Meyer, Gabriele Strauß, Werner Streif, Thomas Thiele, Verena Wiegering, Eva Klopocki, Harald Schulze, on Behalf of the THROMKIDplus Study Group of the Society of Paediatric Oncology Haematology (Gesellschaft für Pädiatrische Onkologie und Hämatologie, GPOH) and the Society of Thrombosis Haemostasis Research (Gesellschaft für Thrombose- und Hämostaseforschung, GTH)

**Affiliations:** 1 University Children's Hospital, University of Würzburg, Würzburg, Germany; 2 Institute of Human Genetics, University of Würzburg, Würzburg, Germany; 3 Centre for Clinical Transfusion Medicine, University Hospital of Tübingen, Tübingen, Germany; 4 Institute for Transfusion Medicine, University of Greifswald, Greifswald, Germany; 5 DRK-Blutspendedienst Baden-Württemberg-Hessen, Institute for Transfusion Medicine and Immunology, Heidelberg University, Mannheim, Germany; 6 University Children's Hospital, Technical University Munich, Munich, Germany; 7 Department of Pediatrics, Carl Gustav Carus University Hospital, Dresden, Germany; 8 Institute of Experimental Biomedicine, University Hospital Würzburg, Würzburg, Germany; 9 Institute for Transfusion Medicine, Charité—Universitätsmedizin Berlin, Berlin, Germany; 10 Department for Pediatric Oncology and Hematology, HELIOS Klinikum Berlin-Buch, Berlin, Germany; 11 Department of Pediatrics, Medical University Innsbruck, Innsbruck, Austria

**Keywords:** next-generation sequencing, molecular genetics, thrombocytopenia, thrombocytopathy, platelet function disorder

## Abstract

Inherited platelet disorders (IPD) form a rare and heterogeneous disease entity that is present in about 8% of patients with non-acquired bleeding diathesis. Identification of the defective cellular pathway is an important criterion for stratifying the patient's individual risk profile and for choosing personalized therapeutic options. While costs of high-throughput sequencing technologies have rapidly declined over the last decade, molecular genetic diagnosis of bleeding and platelet disorders is getting more and more suitable within the diagnostic algorithms. In this study, we developed, verified, and evaluated a targeted, panel-based next-generation sequencing approach comprising 59 genes associated with IPD for a cohort of 38 patients with a history of recurrent bleeding episodes and functionally suspected, but so far genetically undefined IPD. DNA samples from five patients with genetically defined IPD with disease-causing variants in *WAS* , *RBM8A* , *FERMT3* , *P2YR12* , and *MYH9* served as controls during the validation process. In 40% of 35 patients analyzed, we were able to finally detect 15 variants, eight of which were novel, in 11 genes, *ACTN1* , *AP3B1* , *GFI1B* , *HPS1* , *HPS4* , *HPS6* , *MPL* , *MYH9* , *TBXA2R* , *TPM4* , and *TUBB1* , and classified them according to current guidelines. Apart from seven variants of uncertain significance in 11% of patients, nine variants were classified as likely pathogenic or pathogenic providing a molecular diagnosis for 26% of patients. This report also emphasizes on potentials and pitfalls of this tool and prospectively proposes its rational implementation within the diagnostic algorithms of IPD.

## Introduction

Inherited platelet disorders (IPD) form a rare and heterogeneous disease entity that is present in about 8% of patients with non-acquired bleeding diathesis and characteristically associated with mucocutaneous bleeding episodes of variable intensity that may culminate in life-threatening hemorrhage during or after invasive procedures. 1 2 3 Impaired transcription regulation, receptor signaling, cytoskeleton formation, granule composition, or trafficking in megakaryocytes or platelets may result in a reduced circulating platelet count, platelets failing to fulfil their hemostatic function, or even a combined quantitative and qualitative platelet defect. 1 Identification of the defective cellular pathway is an important criterion for stratifying the patient's individual risk profile and for choosing personalized therapeutic options. 4 In addition, precise determination of the gene or pathway involved is substantial for trustworthy genetic counselling of patients and their relatives with respect to the mode of inheritance, recurrence risk, and long-term prognosis, especially in disorders with different inheritance patterns, for example, autosomal dominant Bernard–Soulier syndrome type A2 (OMIM 153670) or severe gray platelet-like disease caused by recently described novel autosomal recessive *GFI1B* variants. 5 6 7

To reach these aims, various diagnostic tools to assess platelet biogenesis and function have been developed over the last decades. 8 Several national and international guidelines have been elaborated to standardize diagnostic processes and laboratory assays. 9 10 11 A recent worldwide laboratory survey of the International Society on Thrombosis and Haemostasis (ISTH), however, revealed that, even if a broad range of analytical methods is applied, the exact underlying defect cannot be identified in more than one-third of patients with confirmed platelet function abnormality. 12 Since knowledge about genes, that are involved in either quantitative or qualitative platelet disorders, has increased rapidly and costs of high-throughput sequencing (HTS) technologies have declined simultaneously over the last decade, molecular genetic diagnosis of bleeding and platelet disorders (BPDs) is getting more and more valuable in diagnostic algorithms. 13 14 15

Several initiatives, primarily from centers in the United Kingdom, have selected and adapted HTS techniques to accelerate the time to diagnose IPD or to identify novel genes or variants involved in the pathogenesis of these rare diseases. The international collaborative ThromboGenomics and BRIDGE-BPD projects have provided a significant impact on research in the field of BPD. While the ThromboGenomics targeted sequencing platform comprises a limited number of initially 63, currently 79 genes with a characterized role in bleeding, thrombotic, or platelet disorders, the BRIDGE-BPD consortium chose a whole exome sequencing (WES) approach that is linked to a standardized phenotype classification, using the human phenotype ontology (HPO), to identify novel genes eliciting a certain phenotype pattern. 16 17 18 19 The United Kingdom Genotyping and Phenotyping of Platelets (GAPP) study has specifically focused on platelets and has identified several novel variants and genes by using HTS techniques. 20 21 22 Just recently, a Scandinavian collaborative group and a Spanish group each published their experiences of diagnosing and classifying BPD by targeted HTS methods and found significant variants in 22 or 68% of the investigated patients, respectively. 23 24

In parallel, members of the German, Austrian, and Swiss THROMKIDplus study group developed, verified, and evaluated a targeted, panel-based next-generation sequencing (NGS) approach for a cohort of 38 patients with a history of recurrent bleeding episodes and functionally suspected, but so far genetically undefined IPD. All detected variants were classified as pathogenic (class 5), likely pathogenic (class 4), uncertain significance (class 3), likely benign (class 2), or benign (class 1). This report presents the results of this study and also emphasizes on potentials and pitfalls of this tool and prospectively proposes its rational implementation within the diagnostic algorithms of IPD.

## Materials and Methods

### Subjects

Thirty-eight unrelated individuals between 1 month and 66 years of age (26 children or adolescents and 12 adults) with suspected IPD were included into the study between January 2015 and January 2016 ( Table 1 ). Each patient had been evaluated in specialized centers in Germany and Austria between June 2007 and January 2016 due to a mild to severe bleeding diathesis according to current guidelines and considered to have a familial, syndromic, or functionally diagnosed platelet disorder with onset in childhood or adolescence. 10 Among these 38 patients whose diagnoses were not genetically defined prior to enrolment, 18 patients (47%) suffered from isolated thrombocytopenia, 16 patients (42%) from a platelet function disorder, and four patients from a combined defect (11%). Among them were four patients highly suggestive to have Hermansky-Pudlak syndrome (HPS) due to impaired platelet function and oculocutaneous albinism. Full blood count and blood smears stained according to May-Grünwald-Giemsa or Pappenheim showed macrothrombocytopenia in six patients (27% of cases with thrombocytopenia). Immunofluorescence microscopy for nonmuscle myosin heavy chain IIa expression was performed to confirm or rule out *MYH9* -associated disorders in indicated cases. 10 25 In the group of platelet function disorders, blood samples were analyzed for plasmatic clotting and von Willebrand factors, Born aggregometry (basically using ADP, epinephrine, collagen, and ristocetin as agonists), and, in some cases, lumino-aggregometry and flow cytometry (mainly to assess Glanzmann thrombasthenia or Bernard–Soulier syndrome) as designated. 10 26 The patient cohort typically reflects various reasons for referral, a broad distribution of treating centers, and a distinct access of even specialized centers to extended diagnostic methods and, thus, provides a rather heterogeneous list of available diagnostic results for this cohort ( Table 1 ).

**Table 1:** Patient characteristics and detected variants classified as classes 3–5 after phase 1 variant assessment (in order of inclusion date)

Case	Age	Sex	Suspected diagnosis and/or referral due to	Platelet count a	Main findings of platelet analysis	Gene	Status b	Detected variant	Segregation	In silico prediction	Reference	Variant class c
1	17 y	m	Suspected HPS; albinism; prolonged traumatic bleeding	210	Abnormal LTA (COL, EPI), CD63 and mepacrine assay (FC)	*HPS6*	hom	c.1919_1920delTC, p.V640Gfs*29	Yes	–	34	4
2	20 mo	m	Familial thrombocytopenia; mucocutaneous bleedings	54	d. n. a.	*ANKRD26*	het	c.127C>G, p.R43G	No	M, S, P	–	2
3	11 y	f	Recurrent epistaxis; hematomas; menorrhagia	171	Abnormal LTA (ADP, EPI, ristocetin), ADP response (FC)							
4	3 y	m	Severe familial thrombocytopenia and bleeding episodes	3	d. n. a.							
5	9 y	f	Severe thrombocytopenia since the age of 2 mo; ITP treatment failure	7	d. n. a.							
6	15 mo	m	Suspected ADP receptor defect; menorrhagia; epistaxis	n. a.	Reduced ADP response (LTA, FC)							
7	4 y	f	Suspected Bernard–Soulier syndrome; thrombocytopenia	n. a.	d. n. a.	*ACTN1*	het	c.1019C>T, p.T340M	n. d.	M, S, P	–	3
8	14 y	m	Suspected ADP receptor defect; joint hemorrhage	230	Abnormal ADP response (LTA, FC), LTA (EPI)							
9	66 y	f	Macrothrombocytopenia; suspected *MYH9* -related disorder	85	Reduced CD62P expression upon TRAP6 (FC)	*TUBB1*	het	c.128–129AG>CC, p.Q43P	n. d.	E, M, S, P	39 40	2 d
10	10 y	f	Suspected HPS; albinism; recurrent epistaxis	375	LTA as aspirin-like defect; delta-granule deficiency (FC)	*HPS4*	hom	c.1714–1G>A (loss of splice acceptor)	Yes	–		5
11	17 y	m	Thrombocytopenia with skeletal abnormalities	102	d. n. a.							
12	14 y	f	Suspected HPS	n. a.	No response upon ADP, TRAP6 (LTA, FC)	*AP3B1*	hom	c.1790delT, p.I597Nfs*3	n. d.	–	30	4
13	13 y	m	Suspected thrombocytopathy; hematomas and petechiae	218	Abnormal LTA (ADP)							
14	18 y	m	Familial, autosomal dominant thrombocytopenia	115	d. n. a.	*MYH9*	het	c.3250_3252delGAG, p.E1084del	n. d.	M	HGMD 35	4
15	14 mo	f	Suspected Glanzmann thrombasthenia	183	Abnormal LTA (ADP, COL); reduced GPIIb/IIIa expression (FC)							
16	10 y	m	Mild familial thrombocytopenia	127	d. n. a.	*TBXA2R*	het	c.343_345delTTC, p.F115del	n. d.	E, M	–	3
17	18 y	f	Suspected aspirin-like defect; menorrhagia	160	Abnormal LTA (ADP, AA, U46619)							
18	12 y	f	Suspected aspirin-like defect	220	Abnormal LTA (ADP, AA, U46619)							
19	13 y	f	Menorrhagia	348	Abnormal LTA (ADP, COL, EPI)							
20	17 y	f	Menorrhagia; hematomas; repetitive postoperative bleeding	363	Giant platelets; abnormal LM (thrombin, COL)							
21	12 y	m	Familial thrombocytopenia	24	d. n. a.							
22	3 y	m	Familial thrombocytopenia	123	Abnormal IF (cytoskeleton, α-/dense-granules)							
23	2 y	m	Familial thrombocytopenia	65	Absent thrombospondin in α-granules (IF)							
24	36 y	f	Severe familial macrothrombocytopenia; platelet function defect	119 e	Abnormal platelet function (LTA), CD34 expression (IF)	*GFI1B*	hom	c.551insG, p.S185Lfs*3	Yes	–	7	4
25	24 y	f	Suspected HPS; albinism	194	Reduced ATP release (COL, EPI), dense-granule content (IF)	*HPS1*	het f	c.598G>T, p.E200*	n. d.	–	–	3 f
						*HPS1*	het f	c.972insC, p.M325Hfs*128	n. d.	E	ClinVar; HGMD 38	3 f
26	32 y	f	Suspected GPVI defect; hypermenorrhea; skin bleedings	280	Abnormal LTA (COL, CVX); reduced response upon CVX (FC)							
27	22 y	f	Familial macrothrombocytopenia; menorrhagia	99	Giant platelets	*ACTN1*	het	c.136C>T, p.R46W	Yes	E, M, S, P	HGMD 36 37	4
28	8 y	f	Macrothrombocytopenia	64	d. n. a.							
29	1 mo	n. a.	Suspected *MYH9* -related disorder; macrothrombocytopenia	83	d. n. a.	*MYH9*	het	c.3464C>T, p.T1155I	n. d.	M, S, P	ClinVar; HGMD 31	5
30	25 y	f	Familial thrombocytopenia	113	Abnormal myosin clustering (IF)							
31	66 y	m	Familial macrothrombocytopenia	85	Abnormal tubulin distribution (IF)	*TUBB1*	hom	c.326G>A; p.G109E	Yes	E, M, S, P	HGMD 32	4
32	8 y	f	Familial thrombocytopenia; skin bleedings after mosquito bites	61	Unclear CD34 expression (IF)	*GFI1B*	het	c.581G>A; p.C194Y	Pat	M, S, P	–	3
						*TPM4*	het	c.385delG; p.V129*	Pat	M	–	3
						*FLNA*	het	c.6047A>G; p.K2016T	Mat	M, S, P	–	2
33	36 y	f	Familial thrombocytopenia; delta-storage pool deficiency	85	Abnormal LTA (ADP, COL, AA), ATP release (LM)							
34	41 y	f	Familial thrombocytopenia	88	Abnormal LTA (AA, ADP, COL, ristocetin)							
35	62 y	m	Thrombocytopenia since the age of 35 y; ITP treatment failure	26	Giant platelets							
36	7 mo	f	Suspected CAMT type II; mucocutaneous bleeding diathesis	<10	n. d. a.	*MPL*	hom	c.769C>T; p.R257C	Yes	E, M, S, P	ClinVar; HGMD 33	5
37	11 y	m	Hematomas; delta-storage pool deficiency	406	Abnormal LTA (COL); reduced ATP and ADP release (LM)							
38	11 y	f	Familial recurrent epistaxis; delta-storage pool deficiency	218	Reduced ATP and ADP release (LM)							

Abbreviations: AA, arachidonic acid; ADP, adenosine diphosphate; CAMT, congenital amegakaryocytic thrombocytopenia; COL, collagen; CVX, convulxin; d. n. a., details not available; E, ExAC; EPI, epinephrine; f, female; FC, flow cytometry; GP, glycoprotein; het, heterozygous; hom, homozygous; IF, immunofluorescence; HPS, Hermansky-Pudlak syndrome; ITP, immune thrombocytopenia; LM, luminometry; LTA, light transmission aggregometry; M, MntTaster; m, male; mat, maternal; n. a., not available; n. d., not done; P, Poly-phen 2; pat, paternal; S, SIFT.

a (× 10 ^9^ /L).

b Allele frequency for heterozygous cut-off: 40–60% of the reads.

c After phase 2 variant assessment.

d The *TUBB1* Q43P variant is not pathogenic on its own, but known to be a modifier of platelet function.

e Under eltrombopag.

f *Trans* -position of heterozygous variants not yet proven, thus, preliminary classification of variants into class 3.

For validation of the targeted, panel-based NGS approach, genomic DNA from five patients between 17 days and 14 years of age with functionally characterized and genetically defined IPD served as controls: adenosine diphosphate (ADP) receptor defect, leukocyte-adhesion deficiency type III, 27 *MYH9* -related macrothrombocytopenia, thrombocytopenia-absent radius (TAR) syndrome, and Wiskott-Aldrich syndrome, respectively ( Supplementary Table S1 ).

### Gene Panel Design

The gene panel was based on causes of quantitative or qualitative IPD that had been published by the time of its compilation in December 2014 and designed using Illumina DesignStudio software (Illumina, San Diego, California, United States). 4 10 28 In contrast to the ThromboGenomics platform or the panel of the Scandinavian collaborative group, genes associated with deficient clotting factors or each type of von Willebrand disease were not taken into consideration, as plasmatic deficiencies are excluded by comprehensive laboratory analysis according to the current underlying guidelines. 10 17 23 For most of the genes, the panel design was restricted to coding sequences. In case of *ANKRD26* and *RBM8A* , which are reported to have variants in the noncoding 5′ untranslated region (5′UTR) or in introns, the design included these noncoding regions. The panel design was supplemented with candidate genes or predisposing genes known from animal models finally resulting in a panel covering 59 genes ( Supplementary Table S1 ).

### Sequencing Procedure and Data Processing

**Fig. 1:**
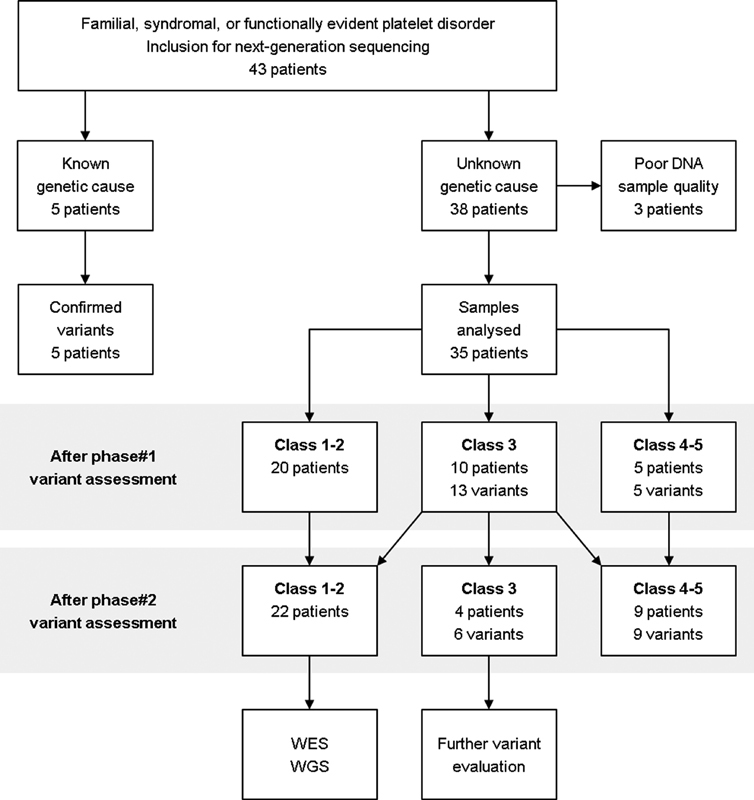
Flow chart of analysis procedure and variant assessment. DNA samples of 43 individuals were processed by targeted, panel-based next-generation sequencing (absolute number of patients). In the group of patients with unknown platelet defect, variants were initially classified using *in silico* filtering programs and gene databases (phase 1 variant assessment). In a second step, class 3 variants (variants of uncertain significance) were assessed with respect to clinical phenotype, segregation analysis, literature, *in vitro* data, and animal models (phase 2 variant assessment). DNA, deoxyribonucleic acid; WES, whole exome sequencing; WGS, whole genome sequencing.

Genomic DNA was isolated from peripheral blood samples of 43 individuals and subjected to targeted, panel-based NGS. Of these 43 individuals, 38 patients had an unknown molecular etiology and 5 patients had well-characterized variants for the validation process ( Fig. 1 ). Target enrichment was performed by Nextera Rapid Capture Enrichment and subsequent sequencing on the MiSeq platform; the MiSeq Reporter was used for the alignment of the sequencing data (Illumina). Three datasets that failed quality control were excluded from further processing and analysis ( Fig. 1 ). The alignments and copy number variations (CNV) of *RBM8A* were analyzed by the GensearchNGS software (version 1.6.58; PhenoSystems SA, Wallonia, Belgium). Alamut Software Suite (version 2.7_1; Interactive Biosoftware, Rouen, France) served as a visualization tool. For variant identification, filters were set as follows: minor allele frequency (MAF) less than 1%, balance of forward and reverse reads greater than 0, and read count greater than 20. Nomenclature has been applied according to Human Genome Variation Society (HGVS; http://hgvs.org/mutnomen ). Variant frequency was checked against ExAC to exclude frequent variants. The detected rare variants were matched with the Human Gene Mutation Database (HGMD) and ClinVar. For variant assessment, the *in silico* prediction programs MutationTaster, SIFT, and PolyPhen-2 as well as splice prediction tools implemented in the Alamut Software Suite (version 2.7_1, Interactive Biosoftware, Rouen, France), that is, Human Splicing Finder (version 3.1) and the NNSplice algorithm ( http://www.fruitfly.org/seq_tools/splice.html ), were used.

In correspondence to the guidelines of the American College of Medical Genetics and Genomics (ACMG) and the Association for Molecular Pathology (AMP) from 2015, detected variants were classified into the five ordinally scaled categories classes 1 to 5. 29 After initial classification using *in silico* filtering programs and gene databases as described (phase 1 variant assessment), variants were classified in a second step with respect to clinical phenotype, segregation analysis, literature, *in vitro* data, and animal models (phase 2 variant assessment).

### Ethical Considerations

Gene sequencing and processing of pseudonymized clinical and laboratory data were conducted at the Institute of Experimental Biomedicine, the University Children's Hospital, and the Institute of Human Genetics of the University of Würzburg between January 2015 and April 2016 in accordance with local Institutional Review Board guidelines and the declaration of Helsinki of 1964 with its later amendments. Written informed consent was obtained from each subject included.

## Results

### Test System Validation

Five DNA samples from patients with functionally characterized and genetically defined IPD served as controls during the validation process. Both sequencing procedure and data processing including *in silico* filtering exactly confirmed the known mutations and CNV in the five genes leading to distinct phenotypes: ADP receptor defect (heterozygous *P2YR12* variant), leukocyte-adhesion deficiency type III (homozygous *FERMT3* variant), *MYH9* -related macrothrombocytopenia (heterozygous *MYH9* variant), TAR syndrome (hemizygous *RBM8A* noncoding intron 1 single nucleotide polymorphism [SNP] and heterozygous *RBM8A* microdeletion), and Wiskott-Aldrich syndrome (hemizygous *WAS* variant), respectively ( Supplementary Table S2 ).

### Gene Panel Analysis

Three out of 38 datasets (cases 21–23) from patients with a so far unidentified genetic cause failed quality control and had thus to be excluded from further analysis. In total, 35 samples reached a mean 20-fold coverage of 97.5% and could be analyzed as described ( Fig. 1 ). Fifteen out of the 35 DNA samples (43%) revealed one or more genetic variants in the tested 59 genes that could be classified as class 3 or higher following *in silico* assessment (phase 1 variant assessment; Fig. 1 ). After phase 1 variant assessment, five variants (14%) in the five distinct genes— *HPS4* (case 10), *AP3B1* (case 12), *MYH9* (case 29), *TUBB1* (case 31), and *MPL* (case 36)—were attributed to class 4 or 5 ( Fig. 1 ; Table 1 ). Ten patients (29%) carried 13 distinct class 3 variants in the genes *HPS6* (case 1), *ANKRD26* (case 2), *ACTN1* (cases 7 and 27), *TUBB1* (case 9), *MYH9* (case 14), *TBXA2R* (case 16), *GFI1B* (cases 24 and 32), *HPS1* (case 25), and *FLNA* and *TPM4* (both case 32; Fig. 1 ; Table 1 ).

No further variant evaluation was required when patients carried class 4 or 5 variants ( Fig. 1 ; Table 1 ). Class 4 or 5 variants in two genes associated with HPS ( *AP3B1* and *HPS4* ) correlated well with the clinical phenotype of the two children (cases 10 and 12). 30 A 1-month-old infant (case 29) and a 66-year-old woman (case 31) with congenital thrombocytopenia were diagnosed with a *MYH9* - or *TUBB1* -related disorder, respectively. 31 32 The known homozygous *MPL* mutation, confirmed by Sanger sequencing, in a 7-month-old infant with severe thrombocytopenia and complete lack of megakaryocytes in the bone marrow aspirate (case 36) provided proof of congenital amegakaryocytic thrombocytopenia. 33

In case of class 3 variants, supplementary clinical and experimental evaluation was needed to further classify the detected variants (phase 2 variant assessment; Fig. 1 ; Table 1 ). Additional functional assays and segregation analysis provided evidence that the novel homozygous frameshift variant in the *HPS6* gene of a 17-year-old adolescent (case 1) is causative for the clinical phenotype of HPS and was thus considered as class 4. 34 Comprehensive genetic investigation was necessary to clear up the pathogenesis of a gray platelet-like syndrome in a family with life-threatening bleeding diathesis (case 24), ending up in the first description of an autosomal recessive *GFI1B* nonsense mutation in the alternatively spliced exon 9, which selectively leads to severe macrothrombocytopenia and platelet dysfunction without critically impairing erythropoiesis. 7 Two other adult patients with familial thrombocytopenia (cases 14 and 27) showed variants in the genes *MYH9* and *ACTN1* that are associated with thrombocytopenia due to impaired megakaryocytic and platelet cytoskeleton regulation according to previously described single reports. 35 36 37 As a consequence, each of these four mentioned variants ( *HPS6* , *GFI1B* , *MYH9* , and *ACTN1* ) could be classified as class 4 variants after phase 2 variant assessment ( Fig. 1 ; Table 1 ). If compound heterozygosity of the two heterozygous variants in the *HPS1* gene in a patient with suspected HPS (case 25) is proven to be in *trans* -position by evaluating both currently unavailable parents, the detected heterozygous variants in combination would also be classified as class 4 ( Table 1 ). 38

In one case of highly suggestive autosomal dominant thrombocytopenia (case 2), the identified but so far undescribed heterozygous variant outside the 5′UTR of the *ANKRD26* gene did not segregate with the phenotype in the family and had thus to be classified as class 2 ( Table 1 ). Since clinical significance of novel heterozygous variants in *ACTN1* and *TBXA2R* (cases 7 and 16) has not yet been completely evaluated, they remained categorized as class 3 variants ( Table 1 ). The heterozygous Q43P substitution in β1-tubulin (case 9) was originally described to modulate platelet function and structure, but is better considered a modifier of platelet function due to its high MAF. 39 40 In consequence, we have now classified this variant as class 2.

Further evaluation becomes more complicated when the clinical phenotype is atypical due to variants in two or more genes: one child (case 32) with moderate thrombocytopenia, skin bleeding tendency, and positive family history (father with moderate thrombocytopenia and recurrent epistaxis; mother with postoperative bleeding and suspected thrombocytopathy) carried a class 3 variant in each of the *GFI1B* , *TPM4* , and *FLNA* genes ( Table 1 ). Cosegregation analysis in this family attributed the autosomal *GFI1B* and *TPM4* variants to the affected father, while the X-chromosomal *FLNA* variant cosegregated with the non-syndromic mother. We reclassified this variant as class 2 ( Table 1 ). Overall, 8 of the 15 variants (53%) classified as classes 3 to 5 after phase 2 assessment were novel ( Fig. 1 ).

After phase 2 variant assessment, 22 out of the 35 samples (63%) did not provide any classes 3 to 5 variant in the investigated genes represented in the panel design ( Fig. 1 ). Of these 22 samples, 10 were from patients with thrombocytopenia and the other 12 from patients with functionally suggestive thrombocytopathy ( Table 1 ). None of the suspected aspirin-like defects (cases 17 and 18) and none of the cases with delta-storage pool deficiency (cases 33, 37, and 38) showed any likely causative variant in the panel genes ( Table 1 ). For all these unexplained cases, an extended genetic analysis by WES or even whole genome sequencing (WGS) may uncover causative variants in novel genes not yet associated with IPD or variants in noncoding sequences (e.g., introns or regulatory elements) that are responsible for the pathogenesis ( Fig. 1 ).

## Discussion

### Potentials in Patient Care and Clinical Research

Our targeted, panel-based NGS approach was sensitive enough to confirm the variants of the five control patients ( Supplementary Table S2 ) including a complete heterozygous loss of the *RBM8A* gene, which is causative for TAR syndrome in a compound-heterozygous state with the predisposing SNP in *RBM8A* . 41 42 In our study cohort of 35 investigated individuals with suspected but so far genetically undefined quantitative or qualitative IPD, we discovered 15 classes 3 to 5 variants ( Fig. 1 ), that may explain the corresponding clinical phenotype, in genes encoding transcription factors ( *n* = 2), cytoskeletal proteins ( *n* = 6), membrane receptors ( *n* = 2), and intracellular vesicle transport regulators ( *n* = 5).

Assuming that the two heterozygous variants in the *HPS1* gene are in *trans* -position (case 25), each of the patient suffering from HPS in our cohort had a class 4 or 5 variant in one of the ten HPS genes. While in the group of 19 patients with isolated or combined thrombocytopenia nine patients (47%) had one or more variants in platelet-associated genes, no variant was detected in the group of 12 patients with solitary, mild to moderate platelet function disorder other than HPS ( Table 1 ). This observation may reflect that we currently do not have sufficient knowledge about all genes involved in platelet function. Of note, some of the bleeding anomalies may be caused by vessel abnormalities or transient interfering factors and are thus not of platelet origin. Finally, we were able to attribute a molecular genetic diagnosis to 9 of the 35 investigated patients (26%) after phase 2 variant assessment ( Fig. 1 ). Overall, the detection rates for known or novel variants in our targeted approach were similar to those recently published by the Scandinavian group. 23

In comparison to sequential exon-by-exon, gene-by-gene Sanger sequencing, HTS methods may improve patient care as comorbidities are diagnosed earlier within regular screening programs: pulmonary fibrosis due to variants in *HPS1* , *HPS4* , or *AP3B1* 43 ; differential impact on platelet count in TAR syndrome 44 ; genotype–phenotype associations in *MYH9* - or *DIAPH1* -related disorders, such as hearing loss, cataract, or renal pathology 45 46 ; or risk of developing leukemia when predisposing genes, such as *ANKRD26* , *ETV6* , or *RUNX1* , are affected. 2 47 In consequence, we consider that it is rational to sequence multiple genes associated with a particular phenotype simultaneously, for example, in case of thrombocytopenia or HPS. 2 43

### Quality Assurance and Pitfalls

HTS methods themselves and the flood of provided genetic data mask several risks or pitfalls: poor coverage of exon 1; false-positive or, to a minor extent, false-negative results during data processing; non-standardized variant classification; or insufficient evaluation of class 3 variants in routine diagnostics. In 2013, the ACMG sets professional clinical laboratory standards for NGS to assure high quality for each step from test development over sample processing to reporting criteria, 48 followed by consensus guidelines of several European societies of human genetics. 49 50 51

Newly established NGS systems must fulfill professional quality standards including assessment of the average read count, the uniformity of coverage, the allelic read percentage, strand biases, quality scores, and types of variants during validation and diagnostic processes. 48 52 In our current panel, only 2.5% of the target sequences had a coverage less than 20-fold. To increase sensitivity of the assay in diagnostic settings, Sanger sequencing is still required to fill gaps in regions with low coverage, especially in GC-rich regions, which are often found in exon 1. Novel techniques aiming to enhance enrichment in these areas may resolve this dilemma to a certain degree. WGS can bypass this problem, but the efforts for data analysis are much higher than for targeted or WES techniques. 53 Besides, NGS approaches may fail to detect CNV, epigenetic phenomena, or mosaicism; so, documentation of quality metrics (e.g., the fraction of 20-fold coverage) is indispensable. 52 Analyses must be repeated when quality parameters have failed (e.g., due to interfering substances or DNA sample impurity as we have encountered in 3 out of 43 samples; Fig. 1 ).

A fundamental element of genetic testing is the interpretation of the detected variants. Several population-based (e.g., ExAC, dbSNP, dbVar, 1,000 genomes), disease-specific (e.g., ClinVar, OMIM, HGMD) or sequence-focused (e.g., NCBI Genome, RefSeqGene) databases as well as *in silico* prediction programs (e.g., SIFT, PolyPhen-2, MutationTaster) serve as a reference for a standardized process of interpretation. In correspondence to the ACMG and AMP guidelines from 2015, variants are currently classified into five categories according to scoring rules that weigh up population-based, *in silico* prediction, functional, segregational, and de novo data. 29 In particular, class 3 variants need further investigation by functional studies, segregation analyses, cell culture assays, or animal models to support the likelihood or unlikelihood that the variant is disease causing. Variants identified by HTS should only be reported in clinical settings for variants affecting established genes causing the specific phenotype of the suspected IPD, also coined “Tier 1 genes.” 54 In addition, genetic counselling of index patients and their relatives is strongly based on the knowledge of *bona fide* identified variants and their mode of inheritance.

The process of variant interpretation is complicated when databases are biased toward a certain ethnical background (e.g., a patient with Asian or Arabic background compared with a Caucasian-biased database) or toward pathogenicity due to the fact that class 1 or 2 variants are reported less frequently to databases, if at all, than class 4 or 5 variants. Furthermore, Freson and Turro evoked convincing concerns that these databases are contaminated with class 1 or 2 variants incorrectly labeled as pathogenic, a circumstance that may lead to a serious feedback loop and, thus, to deceptive care of patients and their relatives. 54

### Prospective of High-Throughput Sequencing in Inherited Platelet Disorders

Since the exact underlying defect cannot be identified in more than one-third of patients with confirmed platelet function abnormality and DNA sequencing costs have rapidly declined over recent years, HTS techniques open up new horizons in this field. 12 13 14 16 In 2015 and 2016 respectively, the Swiss and German public health systems introduced HTS analysis to the list of permitted genetic analyses for diagnostic reasons. By grouping the so far known and relevant genes for suspected pathophysiological mechanisms in platelet biogenesis or function, it becomes possible to have totally or at least partially reimbursed analytical efforts to confirm suspected diagnoses and genetically classify suspected but so far undefined entities of these rare diseases. HTS techniques may also boost scientific activities in the field of IPD, when functional studies, cell culture assays, or animal models allow characterization of novel variants, genes, or pathways. Patients benefit from a notably growing knowledge of these rare hematological diseases, their modes of inheritance, or their particular genetic features, as we could report for one family with severe autosomal recessive gray platelet-like syndrome detected by our NGS panel due to an alternative splice variant in the *GFI1B* gene. 7

The authors consider that the presented NGS gene panel is capable of expanding molecular genetic testing to patients who have failed standard approaches to functionally diagnose or to genetically define IPD according to the published guidelines. 10 11 14 Our experiences and those of international initiatives have been implemented in the study design of the joint patient registry for IPD of the GTH and GPOH with respect to phenotyping by HPO, assurance of comprehensive functional testing, and biomaterial banking. We propose a selective proceeding within the diagnostic algorithm, dependent on the suspected group of IPD (compare Fig. 5 in the study of Andres et al 26 ).
