# Supplementary material for: Use of Targeted High-Throughput Sequencing for Genetic Classification of Patients with Bleeding Diathesis and Suspected Platelet Disorder
Source: TH Open. 2018 Dec 30;2(4):e445–54. doi: 10.1055/s-0038-1676813 (PMC6524924; doi:10.1055/s-0038-1676813)
Supplement: Supplementary file 1 — Supplementary Material [file 10-1055-s-0038-1676813-s180050.pdf]

Use of targeted high-throughput sequencing for genetic classification of patients with bleeding diathesis and suspected platelet disorder

Oliver Andres, Eva-Maria König, Karina Althaus, Tamam Bakchoul, Peter Bugert, Stefan Eber, Ralf Knöfler, Erdmute Kunstmann, Georgi Manukjan, Oliver Meyer, Gabriele Strauß, Werner Streif, Thomas Thiele, Verena Wiegering, Eva Klopocki, and Harald Schulze.

Supplementary Tables

Supplementary Table 1. Panel design of the 59 genes (in alphabetical order within five functional groups).

Functional group	Gene	Transcripts	Mode of inheritance[A]
Transcription regulation	*ANKRD26*[B]	NM_014915.2	AD
	FLI1	NM_001271012.1; NM_001271010.1; NM_002017.4	AD; AR
	GATA1	NM_002049.3	XLR
	GFI1B	NM_004188.6	AD; AR
	HOXA11	NM_005523.5	AD
	JAK2	NM_004972.3	AD
	MPL	NM_005373.2	AR
	RBM8A[B]	NM_005105.4	AR
	ROCK1	NM_005406.2	n. a.
	RUNX1	NM_001754.4; NM_001001890.2; NM_001122607.1	AD
	THPO	NM_001290028.1	AD
Cytoskeleton structure and regulation	*ACTN1*	NM_001130004.1	AD
	DIAPH1	NM_005219.4	AD
	DNM2	NM_001005360.2; NM_001005361.2; NM_004945.3; NM_001005362.2; NM_001190716.1	AD; AR
	FLNA	NM_001110556.1; NM_001456.3	XLD; XLR
	MYH9	NM_002473.5	AD
	PFN1	NM_005022.3	n. a.
	TPM4	NM_001145160.1; NM_003290.2	n. a.
	TUBB1	NM_030773.3	AD
	WAS	NM_000377.2	XLR
Granule biogenesis and trafficking	*AP3B1*	NM_003664.4	AR
	BLOC1S3	NM_212550.4	AR
	BLOC1S6	NM_001311255.1; NM_012388.3	AR
	DTNBP1	NM_032122.4; NM_183040.2; NM_001271668.1; NM_001271669.1	AR
	HPS1	NM_000195.4	AR
	HPS3	NM_032383.4	AR
	HPS4	NM_022081.5	AR
	HPS5	NM_181507.1	AR
	HPS6	NM_024747.5	AR
	LYST	NM_000081.3	AR
	NBEA	NM_015678.4	n. a.
	NBEAL2	NM_015175.2	AR
	STXBP2	NM_001272034.1; NM_006949.3; NM_001127396.2	AR
	VIPAS39	NM_001193314.1; NM_001193316.1	AR
	VPS33B	NM_018668.4	AR

Membrane receptor signalling	*CD36*	NM_001001548.2	AR
	CLEC1B	NM_016509.3	n. a.
	FERMT3	NM_178443.2	AR
	GP1BA	NM_000173.6	AD; AR
	GP1BB	NM_000407.4	AR
	GP5	NM_004488.2	n. a.
	GP6	NM_001083899.2	AR
	GP9	NM_000174.4	AR
	ITGA2B	NM_000419.4	AD; AR
	ITGB3	NM_000212.2	AD; AR
	P2RY1	NM_002563.4	n. a.
	P2RY12	NM_022788.4	AR
	PAR1 (F2R)	NM_001992.4	n. a.
	PLA2G4A	NM_024420.2	n. a.
	SRC	NM_198291.2	AD
	SYK	NM_003177.6	n. a.
	TBXA2R	NM_201636.2; NM_001060.5	AD; AR
Other pathways	*ANO6*	NM_001204803.1; NM_001142679.1; NM_001025356.2; NM_001142678.1	AR
	COL1A1	NM_000088.3	AD
	CYCS	NM_018947.5	AD
	ORAI1	NM_032790.3	AD; AR
	PLAU	NM_001145031.2; NM_002658.4	AD
	PLAUR	NM_002659.3; NM_001005376.2	n. a.
	STIM1	NM_001277961.1; NM_001277962.1	AD; (AR)

[A] Except for somatic mutations; [B] Genes with additional sequencing of non-coding regions.

Abbreviations: AD: autosomal-dominant; AR: autosomal-recessive; n. a.: not available; XLD: X-linked dominant; XLR: X-linked recessive.

Supplementary Table 2. Detection of variants previously identified by Sanger sequencing.

Control	Age	Sex	Diagnosis	Gene	Status	Detected variant	Reference
1	2 m	m	Wiskott-Aldrich syndrome	*WAS*	hem	c.631C>T, p.R211*	
2	5 m	f	TAR syndrome	*RBM8A*	comp het	IVS67+32G>C (first intron TAR SNP) + deletion	(41, 42)
3	17 d	f	Leukocyte-adhesion deficiency type 3	*FERMT3*	hom	p.Q533* (Exon 13)	(27)
4	14 y	f	ADP receptor defect	*P2YR12*	het	c.834T>G, p.Y278*	
5	3 m	m	MYH9-related disorder	*MYH9*	het	c.4270G>A, p.D1424N	

Abbreviations: ADP: adenosine diphosphate; comp het: compound-heterozygous; d: days; f: female; hem: hemizygous; het: heterozygous; hom: homozygous; IVS: intervening sequence; m (age): months; m (sex): male; SNP: single nucleotide polymorphism; TAR: thrombocyto-penia-absent radius syndrome; y: years.
